# The Role of Dyslipidemia in Periodontitis

**DOI:** 10.3390/nu15020300

**Published:** 2023-01-06

**Authors:** Fernando Valentim Bitencourt, Gustavo G. Nascimento, Susilena Arouche Costa, Silvana Regina Perez Orrico, Cecilia Claudia Costa Ribeiro, Fábio Renato Manzolli Leite

**Affiliations:** 1 Department of Dentistry and Oral Health, Section for Periodontology, Aarhus University, 8000 Aarhus, Denmark; 2 Steno Diabetes Center Aarhus, 8200 Aarhus, Denmark; 3 National Dental Centre Singapore, National Dental Research Institute Singapore, Singapore 168938, Singapore; 4 Oral Health Academic Clinical Programme, Duke-NUS Medical School, Singapore 169857, Singapore; 5 Graduate Dentistry Program, Federal University of Maranhão, São Luís 65080805, Brazil; 6 Department of Diagnosis and Surgery, School of Dentistry at Araraquara, São Paulo State University (UNESP), Araraquara 14801385, Brazil; 7 Advanced Research Center in Medicine, Union of the Colleges of the Great Lakes (UNILAGO), São José do Rio Preto 15030070, Brazil

**Keywords:** diabetes mellitus, dyslipidemias, non-communicable diseases, periodontal diseases, public health dentistry, systemic disease

## Abstract

Studies have suggested an important role of dyslipidemia, a condition with alterations in blood lipid levels, in promoting an additional effect on periodontal breakdown. Thus, this study aimed to explore the theoretical pathways associated with dyslipidemia and periodontitis. We used data from 11,917 US adults with complete periodontal examinations participating in the Third National Health and Nutrition Examination Survey (NHANES III). Our hypothesis was tested using structural equation modelling (SEM). Dyslipidemia was defined according to the National Cholesterol Education Program (NCEP-ATP III) and periodontitis as a latent variable reflecting the shared variance of the number of surfaces with periodontal pocket depth [PPD] = 4 mm, PPD = 5 mm, PPD ≥ 6 mm, clinical attachment level [CAL] = 4 mm, CAL = 5mm, CAL ≥ 6 mm, and furcation involvement. The model also considered distal determinants (age, sex, and socioeconomic status) and proximal determinants (HbA1c, smoking and alcohol consumption, and obesity). The model showed sufficient global fit (Root Mean Squared Error of Approximation = 0.04, 90%CI = 0.04–0.05, Tucker–Lewis Index = 0.93, Comparative Fit Index = 0.95). Age, sex, socioeconomic status, obesity, and smoking were directly associated with periodontitis (*p* < 0.01). Dyslipidemia revealed a significant direct effect on periodontitis (standardized coefficient [SC] = 0.086, SE 0.027; *p* < 0.01), also mediated via an indirect pathway through HbA1c (SC = 0.021; SE 0.010; *p* = 0.02) and obesity (SC = 0.036; SE 0.012; *p* < 0.01) and resulted in a total effect on periodontitis. Dyslipidemia was associated with periodontitis through a direct pathway and indirectly through HbA1c and obesity in the US population. These results support the need for a multi-professional approach to tackling oral and noncommunicable diseases (NCDs), directed at their common risk factors.

## 1. Introduction

Noncommunicable diseases (NCDs) contribute substantially to the mortality of 41 million people each year, equivalent to 71% of deaths globally [1]. In the US, approximately 90% of people aged over 65 are affected by at least one NCD, and 73% have two or more, straining health systems [2]. In fact, the heavier the burden of chronic diseases accumulated in an individual, the higher the acceleration into a negative downward spiral of poor self-care and management of the chronic conditions.

Periodontitis is a multifactorial inflammatory NCD that leads to the destruction of tooth-supporting tissues. It is the sixth most prevalent human disease, with its severe form affecting about 10% of the adult population worldwide [3]. In the United States, approximately 50% of adults over 30 years of age are affected by its milder forms [4]. Although preventable, when untreated, severe destruction of the periodontal tissue can progress to tooth loss, commonly leading to masticatory dysfunction and poor nutritional status. A reduction in self-esteem, social interactions, and job performance/opportunities is also observed. Hence, periodontitis negatively impacts the quality of life [4].

An increasing body of epidemiological and experimental studies has evidenced the role that systemic factors, diseases, and conditions may play in the establishment and progression of periodontitis. The mechanisms associated with oral and systemic health encompass shared risk factors and social determinants that alter the immune response locally and systemically, such as genetic and epigenetic factors, acquired risk factors (i.e., socioeconomic status, lifestyle, stress, elevated glucose levels, tobacco and alcohol consumption, and dietary habits high in sugars and fat), pharmaceutical drugs, microbial dysbiosis, and bacteremias [5].

Metabolic changes have been independently associated with periodontitis,^3^ including obesity, hyperglycemia, and abnormal serum lipid profiles [6]. The concomitant occurrence of dyslipidemia in individuals with obesity and diabetes has increased in the US and is reaching epidemic proportions [7]. However, to date, knowledge of the mechanisms and routes involved between dyslipidemia and periodontitis remain scarce, which was the main target of our study. Whether and how lipid abnormalities affect the development of periodontitis through other common pathways needs to be further explored. Observational studies investigating this topic do not present enough power to reach concrete conclusions [6,8]. Therefore, we aimed to explore the pathways between dyslipidemia and periodontitis in a nationally representative sample of US adults from the Third National Health and Nutrition Examination Survey (NHANES III) [9], using a structural equation modelling (SEM) approach.

## 2. Materials and Methods

### 2.1. Data Source and Study Population

This study used data from the NHANES III (1988–1994), conducted by the National Center of Health Statistics (NCHS) of the Center for Disease Control and Prevention (CDC). NHANES III utilized a stratified, clustered, multistage probability sampling design to identify a nationally representative sample of non-institutionalized civilians in the United States.

The total sample was 39,695 subjects over the six years, and 14,421 completed a periodontal examination. We limited the analysis to adults aged 20 and over with a full periodontal assessment (n = 11,917). Detailed descriptions of the survey design, interview, and examinations have been previously published [9]. The NCHS institutional board approved the NHANES III, and all participants provided written informed consent. This study is reported in accordance with the STROBE statement.

### 2.2. Dyslipidemia Assessment

Blood specimens were collected at mobile examination centers and stored appropriately until further analysis. Total cholesterol (TC), high-density lipoprotein cholesterol (HDL-C), low-density lipoprotein cholesterol (LDL-C), and triglycerides (TG) were screened using standard procedures.

The following cutoff values were adopted to classify dyslipidemia according to the National Cholesterol Education Program (NCEP) Adult Treatment Panel-III (ATP-III): triglycerides ≥ 150 mg/dL; total cholesterol > 200 mg/dL; LDL > 100 mg/dL, or HDL < 40 mg/dL in males and 50 mg/dL in females. Participants with ≥ 1 lipid biomarker above the threshold were considered dyslipidemic [10].

### 2.3. Periodontal Examination

Trained dentists performed the periodontal examination according to the NHANES III examination protocol [9]. Briefly, the examination was performed in two quadrants (one upper and one lower) randomly selected at the beginning of the investigation. The buccal and mesiobuccal surfaces, except third molars, were probed separately for each clinical parameter. Thus, the periodontal probing depth (PPD) as the distance in mm from the free gingival margin to the bottom of the sulcus/pocket, and the clinical attachment level (CAL) as the sum of the (a) distance from the free gingival margin to the cement-enamel junction and (b) the distance in mm from the free gingival margin to the base of the pocket, were evaluated in 28 sites. In addition, furcation involvement in the maxillary 1st and 2nd molars, 1st bicuspids, and mandibular 1st and 2nd molars were examined.

The number of sites with PPD equal to 4 mm, equal to 5 mm, and ≥ 6mm, as well as the number of sites with CAL equal to 4 mm, and equal to 5 mm and ≥ 6mm, were recorded. We used periodontal data from NHANES III because it has data on bleeding on probing (BoP) and furcation involvement. However, the variable BoP had a factor loading less than 0.3 for convergent validity, therefore, it was excluded from the latent periodontitis.

### 2.4. Covariates and Serum Measures

Potential confounder covariates for the relationship between dyslipidemia and periodontitis were selected based on a directed acyclic graph. Age, education, sex, income, smoking status, and alcohol consumption were self-reportedly assessed through interviews with structured questionnaires.

Age was grouped as 20–39, 40–59, ≥60 years old; years of education were categorized as ≤8, 9–12, and >12, and sex was classified as male and female. The poverty index (PI) was categorized as ≤1.3, 1.4–3.5, and >3.5 and was calculated by dividing the midpoint of the observed family income by the poverty threshold, the age of the family reference person, and the calendar year in which the family was interviewed [11]. Smoking status was categorized as never, former, and current. The average number of alcoholic beverages consumed per day during the previous year was established as none, light (≤1 drink/day), moderate (1 to 2 drinks/day), and heavy (>2 drinks/day).

Obesity was assessed using the body mass index (BMI) and waist-to-hip ratio. The BMI was calculated as weight (kilograms) divided by squared height (centimeters) and categorized as non-obese when BMI <30 and obese when BMI ≥30. The waist-hip ratio was determined from waist circumference (at the midpoint between the anterior superior iliac crest and the lowest rib) divided by hip circumference (at the level of maximal gluteal protrusion) and categorized as >0.85 for women and >0.90 for men.

Glycated hemoglobin (HbA1c) was measured using the Diamat Analyzer System (Bio-Rad Laboratories, Hercules, CA). According to the American Diabetes Association, HbA1c was dichotomized as <6.5% and ≥6.5% [12].

### 2.5. Statistical Analysis

Descriptive statistics were used to characterize the subjects using weighted frequencies of categorical variables and the mean (± standard error) and 95% confidence interval for continuous variables. Considering the percentage of participants with missing covariates, data were imputed for missing information. Structural equation modeling was performed to explore the association between dyslipidemia and periodontitis using the Mplus Version 8.0 software [13]. SEM is a multivariate analysis that models complex interactions among several predictors, allowing effect decomposition and explicitly identifying direct and indirect relationships.

Initially, a conceptual framework encompassing dyslipidemia as a predictor of poor periodontal status was drawn and hypothesized. Factors related to dyslipidemia and periodontitis were assessed for the possibility of being confounders based on empirical findings and background literature. Subsequently, the theoretical model was carried out according to the conceptual model of the social determinants of health [14]. Firstly, this theoretical model was composed of distal determinants, such as demographic factors (age and sex) [15] and socioeconomic status (education and poverty level) [16]. Secondly, the analysis included proximal determinants as factors related to the general condition and lifestyle behavior (BMI, lipid biomarkers, HbA1c, alcohol consumption, and smoking status) [17,18,19,20,21].

SEM also allows estimating latent variables that are non-observable variables (represented by ellipses in the figures) deduced from the correlation among indicator variables, representing a shared variance of the phenomena to reduce measurement errors [22]. In our theoretical model, the variables SES (socioeconomic status deduced from the correlation between education and poverty index), obesity (inferred from the correlation between BMI and waist-to-hip ratio), and periodontitis (deduced from the correlation between the number of surfaces with PPD = 4 mm, PPD = 5 mm, PPD ≥ 6 mm, CAL = 4 mm, CAL = 5 mm, CAL ≥ 6 mm, and the number of teeth with furcation involvement) were considered as latent variables.

For the SEM analysis, the Weighted Least Squares Mean and Variance Adjusted estimator (WLSMV) was used for continuous and categorical variables and multiple imputations in missing data. Multiple imputation is based on a Bayesian approach and provides unbiased and valid estimates of associations based on information from the available data [22]. The THETA parameterization was used to control differences in residual variances, and STUDYX was used to get standardized coefficients (SC) based on the standard deviation. The following parameters were adopted to assess the goodness-of-fit: (a) Root Mean Square Error of Approximation (RMSEA) with values ranging from zero to 0.08, (b) Comparative Fit Index (CFI) and Tucker–Lewis Index (TLI) with a minimum value of 0.95. Confirmatory factor analysis (CFA) assessed the latent variables concerning SES, obesity, and periodontitis. The latent variable indicators should have factor loadings greater than 0.3 to indicate convergent validity with a *p* value < 0.05 [13].

Additional sensitivity analyses were performed accounting for missing data (Supplemental Table S1), as well as for dietary pattern, by including the Health Eating Index (Supplemental Table S2).

As instructed in the NHANES Manual for Statistical Analysis [9], all analyses considered the study’s complex sampling, sample weight, clusters, and strata to produce generalizable estimates for the US population.

## 3. Results

We limited the analysis to adults aged 20 and over with a full periodontal assessment (n = 11,917). Table 1 shows the weighted characteristics of the study population. According to the NCEP-ATP III, 7586 participants met the dyslipidemia diagnostic criteria (62.20% of the sample). The overall sample’s mean age was 40.04 (SE 25.86, 95% CI: 39.24–40.89 years). The age range was consistently higher among dyslipidemic patients than those without a dyslipidemic component. Statistically significant differences were observed in participants diagnosed with dyslipidemia, such as higher age, alcohol consumption, smoking, BMI, waist-to-hip ratio, HbA1c, and education (*p* < 0.05). However, no significant differences were observed for sex (*p* = 0.11) and PI (*p* = 0.54) (Table 1). Results were consistent in sensitivity analyses accounting for missing data (Supplemental Table S1).

The distribution of the periodontal parameters is displayed in Table 2. Overall, participants presenting dyslipidemia had a higher mean number of sites with PPD = 4 mm, PPD = 5 mm, PPD ≥ 6 mm, CAL = 4 mm, CAL = 5 mm, and CAL ≥ 6 mm compared with those who were without dyslipidemia. Regarding teeth with furcation involvement, no significant differences were observed between subjects with and without dyslipidemia.

The standardized loadings obtained using CFA provided the magnitude of the correlation between the indicators (observed variables) and the latent variable concerning the measurement model. All the loadings for SES, obesity, and periodontitis were statistically significant and substantially high, indicating the appropriateness of the latent variable to represent them (SC ≥ 0.43; *p* < 0.001). Moreover, the measurement model showed adequate global fit (RMSEA = 0.04, 90%CI = 0.04–0.05, TLI = 0.93, CFI = 0.95).

**Figure 1:**
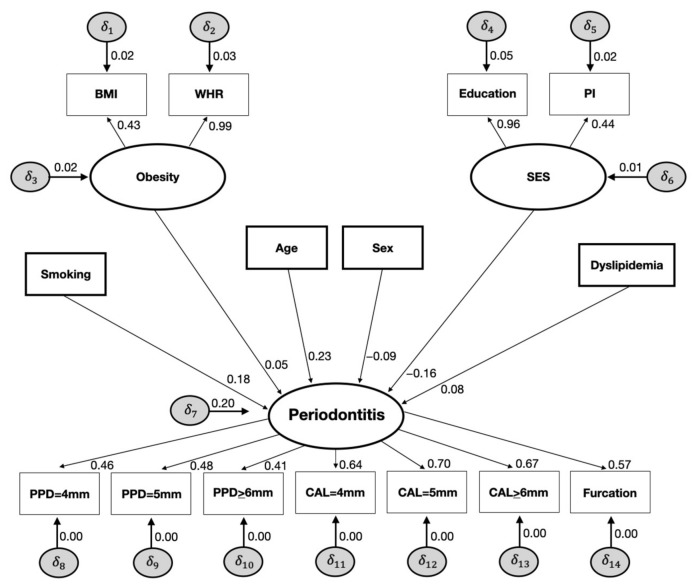
Direct effects of the full structural equation models on the relationships between smoking, obesity, age, sex, SES, dyslipidemia, and periodontitis (NHANES III, 1988–1994).

**Figure 2:**
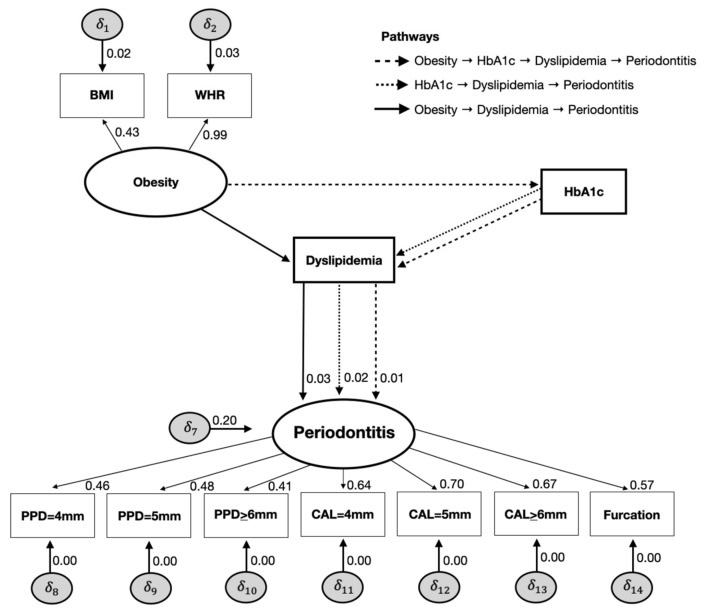
Indirect effects of the full structural equation models on the relationships between obesity, HbA1c, dyslipidemia, and periodontitis (NHANES III, 1988–1994).

The SEM analysis is displayed in Figure 1 and Figure 2. Dyslipidemia was directly associated with periodontitis (SC = 0.086, SE 0.027; *p* < 0.01), as well as obesity (SC = 0.059, SE 0.028; *p* < 0.01), smoking (SC = 0.181, SE 0.015; *p* < 0.01), age (SC = 0.236, SE 0.014; *p* < 0.01), SES (SC = −0.168, SE 0.018; *p* < 0.01), and sex (SC = −0.093; SE 0.012; *p* < 0.01) (Figure 1).

Although the results show a non-statistically significant direct effect of HbA1c on periodontitis (SC = 0.003; SE 0.022; *p* = 0.15), HbA1c exhibited a significant indirect effect on periodontitis mediated by dyslipidemia (SC = 0.021; SE 0.010; *p* = 0.02) and a total effect on periodontal disease (SC = 0.053; SE 0.019; *p* < 0.01). Likewise, when obesity was set as an indirect effect in the SEM, a statistically significant association was found with periodontitis via dyslipidemia (SC = 0.036; SE 0.012; *p* < 0.01). Similarly, there was a statistically significant indirect effect of obesity on HbA1c levels, resulting in dyslipidemia and periodontitis (SC = 0.010; SE 0.002; *p* < 0.01). Accordingly, obesity resulted in a total effect on periodontitis (SC = 0.022; SE 0.022; *p* < 0.01) (Figure 2). Our sensitivity analysis for the dietary pattern revealed a protective effect of a healthy diet on periodontitis (SC = −0.058, SE 0.015; *p* < 0.01), but no changes in the main results were noted (Supplemental Table S2).

## 4. Discussion

In a nationally representative sample of US adults, dyslipidemia was directly associated with periodontitis when socioeconomic status, obesity, and periodontal disease were treated as latent variables, and important confounders were considered in the analytical models. While age, sex, SES, smoking, and obesity were directly associated with periodontitis, no association was observed with HbA1c. However, HbA1c and obesity exhibited a significant indirect and total effect on periodontitis mediated by dyslipidemia, reinforcing the relevance of considering lipid metabolic control in the interrelationship between systemic diseases and periodontitis. To the best of our knowledge, this is the first study using a nationally representative estimate to investigate the pathways between dyslipidemia and periodontitis through SEM.

The literature has shown that SES [16], as well as demographic factors (i.e., age and sex) [15], and factors related to general condition and lifestyle behavior such as BMI [18], lipid biomarkers [21], HbA1c [19], alcohol consumption [20], and smoking status [17] are positively associated with dyslipidemia and periodontitis. In our study, to ensure adequate model fit, all such confounders were incorporated into the analysis for the pathway’s exposure and outcome, strengthening our analytical approach’s robustness.

Periodontitis as a latent variable has been used in other epidemiological studies from our group investigating the association with other NCDs, such as metabolic syndrome [18] and obesity [23]. In addition to reflecting the multidimensional nature of periodontitis, the latent variable also relates well to the classification system proposed in 2017 [4], which defined the primary feature of periodontitis CAL and the proportion of teeth with PPD over certain empirical thresholds. The positive association between dyslipidemia and periodontitis seems biologically plausible in this context. A meta-analysis of observational studies identified a significant association between PPD and abnormalities in the serum lipid levels, e.g., reduction of HDL and the elevation of LDL or TG concentrations [23,24]. However, as most studies did not provide enough periodontal clinical information, a causal interpretation of the findings was undermined.

According to Cekici et al. [25], the relationship between dyslipidemia and periodontitis may result from the systemic inflammatory burden induced by the alteration of serum lipids, which produces high levels of proinflammatory cytokines and alters the host immune response. These effects are influenced by an exacerbated production of advanced glycation end products (AGEs), including low-density lipoprotein (LDL)-AGE and LDL-oxidized, which may act as proinflammatory co-stimulators. With progression, proteins modified by AGE can alter tissue function by cross-linking extracellular matrices. The overproduction of AGEs associated with the elevation of ROS can significantly compromise cellular integrity and cause oxidative damage producing biological effects on lymphocytes and monocytes. Thus, all these elements may connect dyslipidemia and periodontitis.

The findings of this study raise relevant insights into mechanisms that explain the relationship between obesity, diabetes, and dyslipidemia in patients with periodontitis. Prior investigations have clarified at a molecular level how obesity and VLDL can lead to insulin resistance, playing the role of diabetes type 2 precursors. The physiological action of insulin is to directly suppress the production of large VLDL molecules in the liver in analogy to the suppression of glucose production resulting in the overproduction of large VLDL. These results suggest an early phenomenon in hepatic insulin resistance and dysregulation of hepatic lipid metabolism. Additionally, data from animal studies suggest that loss of insulin’s inhibitory action on apoB secretion may be the initial step leading to hypersecretion of VLDL particles [26]. Our findings of dyslipidemia as a mediator of the relationship between diabetes and obesity in periodontitis are also supported by the results of Adiels et al. [27], which found that liver fat, visceral fat, glucose, insulin, and HOMA-IR index are correlated with the rate of VLDL production using a novel multicompartmental model. These observations suggest that obesity and HbA1c appear to be the driving forces behind the overproduction of VLDL1 particles, explaining the indirect relationship between obesity and diabetes in our study. Efforts to address dyslipidemia may help improve these systemic conditions closely related to periodontal disease. As dyslipidemia is a risk factor shared among several chronic diseases, prevention strategies to address NCDs targeting a common risk factor approach may play a major role in periodontitis onset and prevention. Our results suggest that future interventions using a multi-pronged approach to control the level of lipoproteins in the blood are necessary to address obesity and diabetes in individuals with periodontitis.

Nevertheless, in our study, HbA1c revealed a non-statistically significant direct effect on periodontitis. The initial results were intriguing because metabolically decompensated type 2 diabetes mellitus patients present the worst clinical features of periodontitis. Our finding may have been influenced partly by the sample size of participants with HbA1c equal to or higher than 6.5%. In addition, HbA1c was dichotomized according to the American Diabetes Association (2009) [12]. Patients with well-controlled diabetes with HbA1c of about 7% (53 mmol/mol) or less have a limited effect of diabetes on the risk of periodontitis. Nevertheless, the risk increases exponentially as glycaemic control deteriorates, e.g., glycaemic levels above 8%–9% (64–75 mmol/mol) [28]. When HbA1c and obesity were set as potential indirect mediators, a statistically significant association with periodontitis was found to be mediated by dyslipidemia. These results mean that changes in lipid profiles influenced by higher HbA1c levels (defined as ≥6.5%) and obesity (defined by higher BMI and waist-to-hip ratio) subsequently lead to an increased risk of periodontitis. These findings are potentially valuable and indicate a potential mechanism underlying the interrelationship of obesity and diabetes in periodontitis. Moreover, they may indicate that the reduction in HbA1c levels alone may not be enough to decrease the risk of periodontitis if a dyslipidemic profile is observed. This question is still open for future studies.

Our observations align with the World Health Organization and the United Nations’ priorities and strategies to address common NCDs through the Common Risk Factor Approach [29]. Professionals, researchers, and health authorities should be aware of the interplay between lipid level imbalance and exacerbated systemic inflammatory responses to manage the global burden of periodontal diseases effectively. Future research may further elucidate the potential impact of different lipid parameters in obese and diabetic patients on the incidence and fast progression of periodontitis. This is important since the lack of proper systemic management of patients also limits the subsequent rehabilitation process of the oral cavity and the re-establishment of nutritional, esthetic, and psychological elements related to the quality of life.

Although our data indicate a positive direct and indirect effect mediated via dyslipidemia on periodontitis, given the cross-sectional design, the cause-and-effect relationship needs to be considered with caution. While one cannot rule out the possibility of reverse causation, i.e., periodontitis influencing dyslipidemia, one has to bear in mind that all other associations were tested within the current study. If reverse causality were to be a crucial drawback of our study, associations determined by prospective longitudinal studies, such as obesity and periodontitis, would not have been identified. However, the relationship between dyslipidemia and periodontitis should be further investigated in cohort studies accounting for the temporality between presumed exposure and outcome.

There are some limitations to our study. Firstly, the cross-sectional nature of the data makes it impossible to make causal inferences. Secondly, as the current study was carried out on a community level, a full-mouth periodontal protocol examining all teeth with six sites for each tooth was not available. This may have led to underestimating the prevalence of periodontitis in the population. However, several studies have already used these data [30]. Despite its limitations, our study draws upon several strengths. This survey was a representative national sample composed of large sample size, allowing us to control numerous potential confounders and examine direct and indirect interactions between dyslipidemia and periodontitis using SEM.

## 5. Conclusions

In summary, this study indicates that dyslipidemia was associated with periodontitis in the US adult population. Our findings underline the vital role of abnormal serum lipid levels in periodontitis through a direct pathway and indirectly triggered by HbA1c and obesity. This evidence calls attention to the need for a multidimensional approach at all levels of prevention and strategies to address common NCDs, including efforts targeting a common risk factor approach to reduce the burden of dyslipidemia and periodontitis and its complications. Future research can explore the pattern of diet in this relationship. As a recommendation, periodontitis treatment in people living with diabetes should also encompass an evaluation of the management of glucose and triglyceride levels.

## Figures and Tables

**Table 1:** Characteristics of 11,917 US adults with and without dyslipidemia (NHANES III, 1988–1994).

	No Dyslipidemia (n = 2258)		Dyslipidemia (n = 7586)		Missing (n = 2073)		*p*-Value ^2^
	n	% (95% CI) ^1^	n	% (95% CI) ^1^	n	% (95% CI) ^1^	
Age							<0.01 *
20–39	1626	26.34 (24–28)	3608	56.22 (53–59)	1050	17.44 (15–19)	
40–59	418	14.5 (12–16)	2265	68.56 (65–71)	602	16.91 (14–19)	
≥60	214	9.22 (7–11)	1713	73.27 (70–75)	421	17.52 (15–19)	
Sex							0.11
Male	1081	19.24 (17–21)	3570	63.04 (60–65)	1024	17.72 (15–20)	
Female	1177	21.76 (19–23)	4016	61.38 (58–63)	1049	16.87 (15–18)	
Alcohol consumption							<0.01 *
None	920	16.05 (14–17)	3852	67.01 (65–68)	980	16.94 (15–18)	
Light	1098	25.56 (21–27)	2913	57.53 (54–60)	850	17.91 (15–20)	
Moderate	104	18.93 (12–27)	354	68.97 (60–76)	104	12.10 (8–16)	
Heavy	88	18.86 (12–27)	294	60.57 (52–68)	96	20.56 (14–27)	
Missing	48	14.26 (9–21)	173	69.54 (58–78)	43	16.21 (9–26)	
Smoking							0.02 *
Never	1210	21.12 (19–22)	3942	61.15 (58–63)	1079	17.73 (15–19)	
Former	379	18.13 (15–20)	1729	65.17 (62–67)	457	17.70 (14–19)	
Current	669	21.57 (19–24)	1901	61.38 (57–64)	530	17.05 (14–19)	
Missing	---	---	14	87.98 (64–96)	7	12.02 (3–35)	
BMI							<0.01 *
Non-obese	1986	23.71 (22–25)	5309	58.89 (56–61)	1572	17.40 (15–18)	
Obese	270	8.51 (7–9)	2261	74.61 (72–76)	500	16.88 (14–19)	
Missing	2	19.79 (3–6)	16	79.01 (39–95)	1	1.20 (0.1–9)	
Waist-to-hip							<0.01 *
Normal	1273	30.74 (28–33)	2170	51.72 (48–54)	698	17.54 (15–19)	
Above	919	13.21 (11–14)	5133	69.54 (67–71)	1310	17.25 (15–18)	
Missing	66	16.26 (9–25)	283	69.30 (60–76)	65	14.44 (9–20)	
HbA1c							<0.01 *
<6.5%	2195	26.07 (24–27)	6684	73.93 (72–75)	---	---	
≥6.5%	63	7.27	902	92.73 (84–96)	---	---	
Missing	---	---	---		2073	17.29 (15–18)	
Education (years)							0.02 *
≤8	121	15.04 (11–20)	761	70.80 (66–75)	189	14.16- (10–18)	
9–12	338	16.57 (13–19)	1179	65.52 (60–69)	339	17.91 (14–21)	
>12	1783	21.12 (19–22)	5584	61.55 (59–63)	1524	17.33 (15–18)	
Missing	16	11.91 (6–21)	62	72.52 (56–84)	21	15.56 (6–33)	
PI							0.54
≤1.3	704	22.54 (19–26)	2.174	59.51 (55–63)	619	17.95 (15–21)	
1.4–3.5	915	20.04 (18–22)	3.017	63.23 (60–65)	790	16.73 (14–18)	
>3.5	639	20.21 (18–22)	2.395	62.21 (59–65)	661	17.58 (15–19)	

^1^ Weighted percent and 95%CI provided by NCHS. ^2^ *p* values are based on the Chi-square test. * Statistically significant associations *p* < 0.05. Abbreviations: CI, confidence interval; BMI, body mass index; HbA1c, haemoglobin A1c; PI, poverty index.

**Table 2:** Univariate comparisons of periodontal parameters in subjects with and without dyslipidemia (NHANES III, 1988–1994).

	No Dyslipidemia		Dyslipidemia		*p* Value ^2^
	Mean (SE) ^1^	95% CI ^1^	Mean (SE) ^1^	95% CI ^1^	
Periodontal Parameters					
Sites with PPD = 4 mm	0.31 (0.03)	0.23–0.38	0.43 (0.04)	0.34–0.51	<0.01 *
Sites with PPD = 5 mm	0.03 (0.00)	0.02–0.04	0.09 (0.00)	0.08–0.11	<0.01 *
Sites with PPD > 6 mm	0.02 (0.00)	0.00–0.04	0.06 (0.00)	0.05–0.07	<0.01 *
Sites with CAL = 4 mm	0.20 (0.01)	0.16–0.23	0.49 (0.02)	0.44–0.55	<0.01 *
Sites with CAL = 5 mm	0.10 (0.01)	0.07–0.13	0.21 (0.01)	0.19–0.24	<0.01 *
Sites with CAL > 6 mm	0.09 (0.01)	0.06–0.12	0.23 (0.01)	0.19–0.27	<0.01 *
Furcation involvement	0.10 (0.01)	0.07–0.13	−2.27 (2.50)	−7.31–2.76	0.34

^1^ Data are presented as mean with SE and 95% confidence interval provided by NCHS. ^2^ *p* values are based on the Student’s *t*-test. * Statistically significant associations *p* < 0.05. Abbreviations: CI, confidence interval; SE, standard error; PPD, periodontal pocket depth; CAL, clinical attachment loss.

## Data Availability

The data that support the findings of this study are openly available in National Center for Health Statistics at https://wwwn.cdc.gov/nchs/nhanes/nhanes3/ accessed on 3 December 2022.

## Supplementary Materials

The following supporting information can be downloaded at: https://www.mdpi.com/article/10.3390/nu15020300/s1, Table S1: Sensitivity analysis accounting for missing data. Standardized coefficient, standard error, and *p* values of the total and direct effect between dyslipidemia and periodontitis (n = 9844), NHANES III9 (1988–1994). Table S2: Sensitivity analysis considering the Health Eating Index (HEI). Standardized coefficient, standard error, and *p*-values of the total and direct effect between dyslipidemia and periodontitis (n = 9844), NHANES III (1988–1994).
