# Supplementary material for: The Role of Dyslipidemia in Periodontitis
Source: Nutrients. 2023 Jan 6;15(2):300. doi: 10.3390/nu15020300 (PMC9864120; doi:10.3390/nu15020300)
Supplement: Supplementary file 1 [file nutrients-15-00300-s001.zip › nutrients-2086534-supplementary.pdf]

nutrients

MDPI

Supplementary Materials

The Role of Dyslipidemia in Periodontitis

Fernando Valentim Bitencourt [1,2,*], Gustavo G. Nascimento [1,3,4], Susilena Arouche Costa [1,5], Silvana Regina Perez Orrico [6,7], Cecilia Claudia Costa Ribeiro [5] and Fábio Renato Manzolli Leite [1,3,4]

Table S1. Sensitivity analysis accounting for missing data. Standardized coefficient, standard error, and p-values of the total and direct effect between dyslipidemia and periodontitis (n = 9844), NHANES III[9] (1988–1994).

Variables	Effect	Periodontitis		
		β	**SE**	**P-value**
Dyslipidemia	Direct	**0.037**	**0.012**	**0.001**
HbA1c	Total	**0.022**	**0.010**	**0.021**
	Direct	0.013	0.011	0.233
	Indirect			
	Glycohemoglobin-Dyslipidemia-Perio	**0.009**	**0.004**	**0.033**
Obesity	Total	**0.105**	**0.024**	**<0.001**
	Direct	**0.056**	**0.029**	**0.054**
	Indirect			
	Obesity- Dyslipidemia-Perio	**0.036**	**0.012**	**0.002**
	Obesity- Glycohemoglobin-Dyslipidemia-Perio	**0.005**	**0.002**	**0.035**

Significant p-values are exposed in bold. β- Standard coefficient; SE- Standard Error.

Table S2. Sensitivity analysis considering the Health Eating Index (HEI). Standardized coefficient, standard error, and p-values of the total and direct effect between dyslipidemia and periodontitis (n = 9844), NHANES III (1988-1994).

Variables	Effect	Periodontitis		
		β	**SE**	**P-value**
Dyslipidemia	Direct	**0.103**	**0.026**	**<0.001**
HbA1c	Total	0.031	0.019	0.110
	Direct	0.006	0.023	0.794
	Indirect			
	Glycohemoglobin-Dyslipidemia-Perio	**0.024**	**0.011**	**0.026**
Obesity	Total	**0.082**	**0.021**	**<0.001**
	Direct	0.032	0.026	0.221
	Indirect			
	Obesity- Dyslipidemia-Perio	**0.042**	**0.011**	**<0.001**
	Obesity- Glycohemoglobin-Dyslipidemia-Perio	**0.006**	**0.003**	**0.033**

Significant p-values are exposed in bold. β- Standard coefficient; SE- Standard Error.
